# Investigation of metal concentration distribution and corresponding health exposure assessment of fabricated metal product manufacturers

**DOI:** 10.1038/s41598-024-64277-0

**Published:** 2024-06-13

**Authors:** Cheng-Hang Lan, Lun-Chun Ou, Hung-Hsin Liu, Chiung-Yu Peng

**Affiliations:** 1 Department of Occupational Safety and Health, Chung-Hwa University of Medical Technology, Tainan, 71703 Taiwan; 2 Department of Public Health, College of Health Sciences, Kaohsiung Medical University, 100 Shih-Chuan 1st Rd, Kaohsiung, 80708 Taiwan; 3 Department of Occupational Safety and Health, Chung Shan Medical University and Chung Shan Medical University Hospital, Taichung, 40201 Taiwan; 4 Research Center for Precision Environmental Medicine, Kaohsiung Medical University, 100 Shih-Chuan 1st Rd, Kaohsiung, Taiwan; 5 Department of Community Medicine, Kaohsiung Medical University Hospital, 100, Tzyou 1st Road, Kaohsiung, Taiwan

**Keywords:** Health hazard, Hazard index, Incremental lifetime cancer risk, Surface treatment plants, Environmental sciences, Risk factors, Chemistry

## Abstract

The fabricated metal product industries were identified as producers of variable and heterogeneous pollution. Workers in these manufacturing facilities are exposed to multiple pollutants present at variable concentrations. Specific known adverse health effects include bladder cancer associated with metalworking fluid exposure and lung cancer associated with electroplating processes. To reduce the incidence of these adverse effects, the main challenge is to identify the most hazardous pollutants within this complex exposure environment and evaluate the corresponding health potentials. In this study, exposure indices were formulated to assess multiple metal exposures with the ultimate goal of providing relevant information for exposure reduction and control measures. Fifteen plants, including metal mold manufacturing, metal casting, and surface treatment plants, were investigated in terms of total concentration, summation of corresponding ratio to threshold limit value (STLV_r_), hazard index (HI), and incremental cancer risk. The results revealed that emissions of aluminum, iron, and manganese were primarily found in the metal mold manufacturing/casting plants, while emissions of chromium, nickel, and zinc were found in surface treatment plants. STLV_r_ and HI were more useful than the total concentration for identifying hazardous metals, which were chromium and nickel, and could specify the facilities that were in need of control measures. As for cancer risk, the metal mold manufacturing/casting plants had lower risk than the surface treatment plants, and the contributing metals for these two plant types were cobalt and chromium, respectively. This study established a useful procedure to evaluate health hazards and cancer risk. The resulting information is useful for prioritizing mitigation control of multiple metal exposures.

## Introduction

According to statistics from the Taiwan Ministry of Economic Affairs in 2021, the fabricated metal product manufacturing sector comprised 43,691 manufacturers, employed 404,011 workers, and generated a production value of NT$1679 billion, accounting for 26.5%, 13.0%, and 4.9% of the manufacturing industry, respectively^1^. The fabricated metal product industry encompasses several industrial groups, including cutlery and hand tool manufacturing, metal surface finishing, metal mold and die manufacturing, metal casting and forging, metal structures and architectural component manufacturing, and screw, nut, and rivet manufacturing. Despite being collectively categorized within a specific industrial sector, the manufacturing activities are diverse. Consequently, hazardous factors are variable and heterogeneous, posing a challenge in identifying the hazardous factors in this industry sector and the corresponding adverse health effects related to exposure.

Several occupational diseases have been reported in fabricated metal product manufacturing, including the association of metalworking fluid exposure with bladder cancer^2^, and the association of metal plating with lung cancer^3^. A study on the burden of occupational cancer in Britain estimated using attributable fractions, reporting the top three cancers attributable to the metal worker sector as non-melanoma skin cancer (778/1252, cancer cases in this cancer site/total cases in this sector), bladder cancer (252/1252), and lung cancer (174/1252), with the possible carcinogen being mineral oils. The same study also indicated that lung cancer was the predominant cancer in the fabricated metal product sector, with attributable cancer registrations of 68 out of 94 total cases, and the possible carcinogens being chromium VI (Cr(VI)), acid mist, and silica. Therefore, it concluded that lung cancer was the prevalent cancer in metal-related sectors^4^.

Workers are often exposed to multiple pollutants predominantly through inhalation. For instance, Thornéus et al. reported air concentration levels of several metalworking fluid-related pollutants, including dust (0.14–0.94 mg/m^3^), oil mist (0.20–1.20 mg/m^3^), formaldehyde (0.02–0.08 mg/m^3^), and monoethanolamine (0.005–0.16 mg/m^3^). Decreases in exposure to these pollutants were significantly associated with the mitigation of respiratory irritation symptoms such as stuffy or runny nose, dry throat, and cough^5^. Another example is the electroplating process, which commonly involves acid cleaning, nickel plating, and chromium plating, exposing workers to sulfuric acid mist, nickel (Ni), and chromium (Cr) compounds with possible concentration levels of 0.01–7.3 mg/m^3^, 0.76–32.0 μg/m^3^, and 1.2–26.2 μg/m^3^, respectively^6,7^. Die and mold manufacturing plants as well as metal casting plants also pose several hazards. For instance, Sivapirakasam et al. reported concentrations of iron (Fe), Cr, copper (Cu), and hydrocarbons during the electrical discharge machining process in a die and mold manufacturing plant, with corresponding levels of 0.28–3.08 mg/m^3^, 0.047–0.538 mg/m^3^, 0.002–0.107 mg/m^3^, and 0.058–0.814 mg/m^3^, respectively^8^. Peixe et al. documented mean concentration ranges of lead (Pb), cadmium (Cd), and manganese (Mn) in three nonferrous metal foundries, which were 15.37–19.26 μg/m^3^, 7.07–9.14 μg/m^3^, and 8.83–16 μg/m^3^, respectively^9^.

The emitted metal composition from the fabricated metal product industry sector depends on the manufacturing processes and materials used. For example, the emitted metals in electroplating plants depend on the intended plating metals. The most commonly used deposited metals were zinc (Zn) (about 15%), followed by Ni, Cu, Cr, and tin (Sn)^10^. As for metal mold manufacturing plants, they involve processes such as computerized numerical control (CNC) milling, welding, and grinding; therefore, base metal materials such as Fe, aluminum (Al), Mn, and Cu are abundant metals^8,11^. Similarly, metal casting plants involve processes like metal melting, sand molding, and finishing, which result in metal fume and dust of base metals such as Fe, Al, Cu, and Zn^12^. Among these metals, Cr, Ni, and Mn exert considerable adverse health effects^13–17^.

Cr is extensively utilized in various industries due to its exceptional resistance to corrosion and hardness, being a transition metal with common oxidation states including 0, II, III, and VI. Both Cr(III) and Cr(VI) are significant health concerns due to their widespread presence and adverse effects. While Cr(III) is considered less toxic, it can still inflict damage to cell organelles upon entering cells through pinocytosis^13^. Conversely, Cr(VI) has been classified by the International Agency for Research on Cancer (IARC) as a Group 1 carcinogen^14^, linked to various acute and chronic health issues such as cancer, dermatitis, asthma, bronchitis, hypertension, chromosomal aberrations, and DNA damage in lymphocytes^15^. Ni also poses serious health risks, with soluble and insoluble Ni compounds categorized as Group 1 carcinogens, and Ni alloys classified as Group 2B carcinogens by IARC. Exposure to Ni, especially through inhalation, carries significant risks including cardiovascular disease, asthma, lung fibrosis, nasal irritation, nasal septum perforation, and respiratory tract cancers. Skin absorption can also lead to allergic contact dermatitis, particularly among workers handling stainless steel and nickel-plated items^16^. Mn, while essential in trace amounts, can cause adverse health effects when exposure exceeds normal levels. Inhalation or ingestion of excess Mn can lead to dose-dependent nervous system pathology. Individuals exposed to concentrations twenty thousand times higher than those normally found in the environment may experience symptoms like slow hand movement, loss of coordination and balance, forgetfulness, anxiety, or insomnia. Workers exposed to a million times higher manganese concentrations compared to normal air concentrations may develop manganism, characterized by tremors, difficulty walking, and facial muscle spasms. Additionally, lung toxicity due to Mn exposure may result in inflammatory responses and increased susceptibility to infections, potentially leading to manganic pneumonia^17^ (Detailed information in Supplementary information).

Several studies have indicated that co-exposure to multiple metals may result in additive or multiplicative health effects compared to single compound exposure. An analysis of the 1999–2006 NHANES data showed that the odds of increased prevalence of albuminuria and reduced estimated glomerular filtration rate (eGFR) were fourfold higher in participants in the highest quartiles of both Cd and Pb exposure compared to those in the lowest quartile^18^. McDermott et al.^19^ investigated the association of intellectual disability (ID) in children and co-exposure to Pb and arsenic (As) during the prenatal period. The probability of ID was nearly sixfold higher when children were exposed to As at 22 mg/kg and Pb at 400 mg/kg compared to those exposed to As at 22 mg/kg and Pb at 200 mg/kg. A subsequent review provided a summary of the effect of co-exposure to Pb and Mn on pediatric neurodevelopment^20^, concluding that Mn may exacerbate the neurotoxic effects of Pb, especially in younger children. The aforementioned studies suggest that populations with co-exposure to multiple metals might be at increased risk for adverse health effects.

There are numerous subsectors within the fabricated metal product industry, each presenting complex and variable hazards in terms of species and quantity due to the diverse types of raw materials used. Consequently, workers in this industry are exposed to multiple pollutants spanning a wide range of concentrations, posing a significant challenge that necessitates thorough exposure assessment. To address this issue, the objectives of this study were to quantify the types and concentrations of metal distribution in the fabricated metal product industry and to formulate several exposure indices for evaluating the health hazards associated with multiple metal exposures. The resulting information could serve as the foundation for implementing appropriate exposure reduction and control measures.

## Methods

### Description of selected manufacturers

Fifteen fabricated metal manufacturers were recruited, comprising seven surface treatment plants, five metal mold manufacturing plants (involved in CNC milling operation), and three metal casting plants (usually involved in aluminum-related products) located in Taiwan. Detailed information about these plants is provided in Table S1. The selection of manufacturers was based on their distribution across the Taiwan region^1^, with a distribution ratio of 1:2:1 for the northern, central, and southern regions, respectively. Therefore, cluster sampling was employed to ensure that the distribution ratio was met, thus obtaining distribution representative samples.

The three types of manufacturing plants are described as follows:

#### Surface treatment plants

Surface treatment includes electroplating and anodizing procedures. Electroplating processes typically involve several basic steps, including degreasing, rinsing, acid pickling/activation, electro-cleaning, electroplating, baking, passivation, top coating, and drying. During the process, a metal film is deposited on a solid substrate by the electrical reduction of cations of the corresponding metal. The commonly used metals are Zn and zinc alloys, followed by Ni, Cu, and Cr^10^.

Anodizing processes include blasting/etching, rinsing, anodizing, coloring, and sealing. Anodizing follows a similar procedure to electroplating, except that the treated part serves as the anode electrode through which electric current passes to form an oxide surface layer. Anodizing treatment is most commonly used for aluminum alloys, but it is also applicable to other materials such as titanium, zinc, tantalum, and niobium^21^.

#### Metal die and mold manufacturing plant

The processes involved in metal die and mold manufacturing include mold design, CNC milling, electrical discharge machining, polishing, welding, and assembling. During these processes, metal dust and fumes are generated, with the common metal species being Fe, Al, and Zn^8,11^.

#### Metal casting plants

Metal casting plants typically involve several basic steps, including mold preparation, metal melting, pouring, casting, mold cooling, and finishing. The predominant metal hazards in casting processes are dust and fumes, primarily arising from Fe and Al^12^.

### Sampling location and period

Total particulate matter was sampled using a 37-mm cassette holder (closed-face) with a mixed cellulose ester (MCE) filter at a flow rate of 3.5 L/min for at least six hours, following National Institute for Occupational Safety and Health (NIOSH) Method 7302^22^. The flow rate was calibrated before and after sampling, with a variation within 5%. Both area and personal samples were collected. Area samples were placed at fixed locations in each plant, typically consisting of 4 to 8 samples, with one located in the administrative area and the remainder in the manufacturing area. Personal samples were attached to workers’ collars to evaluate personal exposure, with five samples taken in each plant: one from an administrative staff member and the rest from workers in the manufacturing area. The sample size for area and personal samples was 86 and 75, respectively. For each sampling campaign, 10% or at least two field blanks were taken. This study was approved by the institutional review board of Kaohsiung Medical University Hospital (KMUHIRB-E(1)20180247) and conducted according to the Declaration of Helsinki.

### Sample analysis

After sampling, filters were assessed for ten metals: aluminum (Al), cadmium (Cd), cobalt (Co), chromium (Cr), copper (Cu), manganese (Mn), molybdenum (Mo), iron (Fe), nickel (Ni), and zinc (Zn). The filters were decomposed with a 10-mL digesting solution (8 mL of 68% HNO_3_ and 2 mL of 30% H_2_O_2_) in a microwave digestion system (MARSXpress CEM, Matthews, North Carolina). The extract was diluted to 25 mL with 0.2% HNO_3_ and analyzed by an inductively coupled plasma optical emission spectrometer (ICP-OES, Model: Optima 2100DV, PerkinElmer, Shelton, Connecticut)^23^. This analysis method can detect metal elements in various forms of speciation and express them as total element concentrations for a particular element, but it does not determine the metal speciation. Supplemental Table S2 shows quality assurance/quality control (QA/QC) data for these ten metals. Linearity ranged from 0.9971 to 0.9999. The recoveries ranged from 97.9% to 103.4%. The method detection limits (MDL) ranged from 2.14 × 10^–4^ to 1.92 × 10^–2^ μg/m^3^ with respect to sampling conditions in the field. The blank values ranged from non-detectable (ND) to 0.551 μg/sample for medium blanks.

### Health hazard and chronic health effect assessment of multiple metal exposure

#### Application of the additive mixture formula

According to the guidance of the threshold limit values (TLV) developed by the American Conference of Governmental Industrial Hygienists (ACGIH)^24^, when two or more hazardous substances do not show synergistic or antagonistic effects, the additive effect should be assumed. The additive formula was applied to assess the health effects as follows.

1 $${TLV}_{r}= \frac{C}{T},$$

2 $${STLV}_{r }={TLV}_{r1 }+{TLV}_{r2 }+\dots +{TLV}_{rn },$$

where C (μg/m^3^) indicates the observed atmospheric concentrations of the specific pollutant, and T (μg/m^3^) is the corresponding threshold limit. TLV_r1_, TLV_r2_, … TLV_rn_ are the ratios of the observed concentrations to the corresponding threshold limits, and STLV_r_ reflects the summation of TLV_rn_. If STLV_r_ is greater than unity, the threshold limit of the mixture should be considered exceeded, potentially posing health hazards. Since TLVs are guidelines used by industrial professionals for evaluating and controlling potential workplace health hazards, this method was applied to estimate the health hazards of multiple metal exposures and determine the dominant pollutant contribution for this ratio.

#### Hazard quotient and hazard index

The similar approach developed by the California Environmental Protection Agency for non-cancer chronic effect was used. The reference concentration (REF_c_, μg/m^3^) was used to evaluate the hazard quotient (HQ) as expressed in Eq. (3):

3 $$\text{HQ}=\frac{C}{{REF}_{c}},$$

where C is the sample concentration (μg/m^3^). Furthermore, the sum of hazard quotients (denoted as hazard index, HI) of compounds, which are aimed at the same organ, is also assessed using the following equation:

4 $$\text{HI}=\Sigma \frac{{C}_{i}}{{REF}_{c}}.$$

### Estimating incremental lifetime *cancer* risk (ILCR)

The cancer risk of workers was estimated according to the risk assessment guidelines established by the California Environmental Protection Agency^25^, a method widely used in the literature^26,27^. The guideline formula for the incremental lifetime cancer risk (ILCR) is:

5 $${\text{ILCR }} = \, ({\text{C}} \cdot {\text{IR}} \cdot {\text{ET}} \cdot {\text{EF}} \cdot {\text{ED}} \cdot {\text{PF}})/({\text{BW}} \cdot {\text{LT}}),$$

where C is metal concentration (μg/m^3^), IR is inhalation rate (0.83 m^3^/h), ET is exposure time (8 h/day), EF is exposure frequency (250 days/year)^25,28^, ED is exposure duration (40 years, i.e. age 25 to 65 years), BW is body weight (69.2 kg^27^ for males in Taiwan), LT is lifetime (77.7 years for male in Taiwan^29^), and PF is cancer potency factor ([μg/kg/day]^–^^1^). Among the ten metals, Cd, Co, Cr and Ni are associated with carcinogenicity. The potency factors for these metals were 0.015, 0.027, 0.51 and 0.00091, respectively^25^. The sum of ILCR of these four metals was calculated to quantify the cancer risk in each plant. The regulatory limits, recommended limits, and exposure assessment parameters are shown in supplemental information Table S3.

### Data analysis

Data analysis was conducted using Microsoft Excel and IBM SPSS Statistics 22 software (IBM SPSS Inc., Armonk, NY, USA). The regression on order statists (ROS) method, implemented through ProUCL version 5.2.00 (USEPA), was utilized to estimate non-detected values (NDs) for metals with censoring rates < 50%, as a censoring rate greater than 50% could introduce substantial bias^30^. These specific metals were used to assess statistical significance and exposure. The NDs rate (or censoring rate) was classified into three categories: low (< 30%), moderate (30%—50%), and high (> 50%)^30^. The non-parametric Mann–Whitney test was employed to compare metal concentrations between area and personal samples, manufacturing and administrative sites, and metal mold manufacturing/casting plants and surface treatment plants. This statistical test is used to determine whether there is a difference between two groups by the rank sums of the two samples. Briefly, all observations are ranked in order of magnitude, the rankings of two groups are calculated, and the U values of two groups are computed using Eqs. (6), (7). The smaller value of U is used to calculate the Z score, and then the p-value for a given z-value can be obtained^31^.

6 $${U}_{1}={n}_{1 }{n}_{2} +\frac{{n}_{1 }\left({n}_{1 }+1\right) }{2} -{R}_{1},$$

7 $${U}_{2}={n}_{1 }{n}_{2} +\frac{{n}_{2 }\left({n}_{2 }+1\right) }{2} -{R}_{2},$$

8 $$U=\text{min}\left({U}_{1 },{U}_{2 }\right),$$

9 $${U}_{mean}={u}_{U}= \frac{{n}_{1 }{n}_{2}}{2},$$

10 $${\sigma }_{U}= \sqrt{\frac{{n}_{1 }{n}_{2}({n}_{1 }+{n}_{2}+1)}{12},}$$

11 $$z=\frac{U-{u}_{U}}{{\sigma }_{U}},$$

where n_1_ and n_2_ are the observations of the 1st and 2nd populations, R_1_ and R_2_ are the sums of the observation ranks for the 1st and 2nd populations, U_1_ and U_2_ are the U statistics for the 1st and 2nd populations, u_U_ and σ_U_ are the mean and standard error of U, respectively. Statistically significant differences were identified using a two-tailed p-value of < 0.05, with the null hypothesis considering no difference between two comparison groups. Minimum sample size of 22 for each group was determined using G*Power 3.1^32^ with a type I error of 0.05, a power of 0.8 and an effect size of 0.9 (the average value in this study). Since concentrations, chronic health exposure indices, and incremental cancer risk were estimated values, three significant figures were applied based on the accuracy of measurements.

### Informed consent

The study was conducted in accordance with the Declaration of Helsinki and approved by the Institutional Review Board at Kaohsiung Medical University Hospital (KMUHIRB-E(1)20180247). Informed consent was obtained from all subjects.

## Results

### Area and personal concentration distribution

The distribution of area and personal concentrations is presented in supplemental information Tables S4, S5. For area samples (Table S4), the censoring rates varied widely. Metals such as Al, Cr, Cu, Fe, Mn, and Zn had censoring rates of less than 30%, while Ni had a censoring rate of 48%. However, Cd, Co, and Mo had censoring rates exceeding 50%. All metals exhibited a wide distribution of concentrations, as indicated by the coefficient of variance (CV), ranging from 136 to 723%. The variety and quantity of metals found in the investigated plants were diverse. For instance, Ni exhibited a moderate ND rate with a median concentration of 0.0267 μg/m^3^, yet a high concentration of 298 μg/m^3^ was observed in area sampling. This suggests varying usage levels of Ni among manufacturers. Typically, base metals like Cu, Fe, and Al showed the highest concentrations at 1308, 509, and 153 μg/m^3^, respectively.

A similar trend was observed for personal samples. The group with a low censoring rate included Al, Cr, Cu, Fe, Mn, and Zn, while Ni was in the moderate censoring rate group, and Cd, Co, and Mo were in the high censoring rate group. A wide range of concentrations was observed for all metals, with CVs ranging from 91 to 584%. Notably, Al, Fe, and Mn, which were major constituents in metal products, exhibited the highest concentrations at 211, 1200, and 22.5 μg/m^3^, respectively (Table S5).

Comparisons were made between area and personal samples (Table 1) for metals with ND rates below 50%. The mean and median pairs for Al, Cr, Cu, Fe, Mn, Ni, and Zn were 6.25 and 0.387, 0.586 and 0.0658, 18.5 and 0.0972, 12.9 and 2.18, 0.470 and 0.0511, 12.5 and 0.0267, 1.95 and 1.08 μg/m^3^ in area samples, respectively. The corresponding values for personal samples were 6.12 and 0.443, 0.255 and 0.0546, 0.439 and 0.137, 23.9 and 2.05, 0.643 and 0.0521, 0.612 and 0.0332, 1.52 and 0.899 μg/m^3^. Mean values were notably higher than median values, indicating a broad and right-skewed distribution of concentrations among these facilities. Additionally, both area and personal samples exhibited similar magnitudes in mean and median values, indicating no significant differences between them according to the Mann–Whitney test.

**Table 1:** Concentration (μg/m^3^) of area and personal samples in 15 fabricated metal product plants.

Metal	Area (N = 86)					Personal (N = 75)					p value
	Mean	(SE)	Median	Min	Max	Mean	(SE)	Median	Min	Max	
Al	6.25	(2.53)	0.387	0.005	153	6.12	(2.91)	0.443	0.016	211	0.357
Cr	0.586	(0.320)	0.0658	0.0003	27.1	0.255	(0.069)	0.0546	0.0002	2.89	0.885
Cu	18.5	(16.2)	0.0972	0.0015	1380	0.439	(0.130)	0.137	0.001	8.14	0.388
Fe	12.9	(6.31)	2.18	0.05	509	23.9	(16.4)	2.05	0.07	1200	0.855
Mn	0.470	(0.190)	0.0511	0.0007	14.0	0.643	(0.323)	0.0521	0.0012	22.5	0.887
Ni	12.5	(5.56)	0.0267	0.00001	298	0.612	(0.197)	0.0332	0.000003	10.8	0.928
Zn	1.95	(0.31)	1.08	0.02	14.3	1.52	(0.26)	0.899	0.038	12.7	0.565

### The rationale of application of an additive formula for health hazard estimation

In this study, health hazards and non-carcinogenic chronic effects were estimated by calculating the summation of ratios for individual compound levels to their corresponding TLVs and reference doses, respectively. This application assumed that the combined biological effect of the components is equal to the sum of each agent given alone when two or more chemicals have similar adverse health effects on the same organ or system and do not indicate synergy or antagonism effects^24^. Therefore, the health effects of the investigated metals were scrutinized to examine whether this assumption was fulfilled.

Metals such as Al, Cd, Cr, Cu, Fe, Mn, Ni, and Zn, which had ND rates of less than 50%, were included in estimating health effect potentials. All these metals showed certain adverse health effects on the respiratory system, manifested as irritation, coughing, dyspnea, abnormal chest X-rays, emphysema, bronchitis, pneumoconiosis, pneumonia, asthma, and even lung and nasal cancers^15,16,33–39^. This provides supportive information for using the additive formula to estimate adverse health effects of multiple metal exposures.

The HQ method and similar approaches neglect different mechanisms of action of multiple metal exposure and their possible interactions, such as antagonistic effects, synergistic effects or even distinct effects that each metal alone would not exhibit^38^. This is an inherent challenge in investigating health effects of combined exposure. Further studies of in-vitro, in-vivo, and epidemiology are needed to clarify the relationship and mechanism between health effects and multiple exposures. In addition to adverse health effects on the respiratory system, some other effects, such as neuropsychological disorders (due to inhalation exposure to Al and Mn), DNA damage (due to inhalation exposure to Cr and Ni), kidney damage (due to exposure to Cd), and dermatitis (due to dermal exposure to Cu and Ni), were found^15,16,33–39^. This indicates that our estimation may still underestimate the effect of multiple exposures. Nevertheless, the HI method can play a role as a rapid screening tool for multiple pollutant exposure.

### Manufacturing and administrative site concentration comparison

In examining the difference between manufacturing and administrative sites (Table 2), comparisons were made for metals with censoring rates below 50%, including Al, Cr, Cu, Fe, Mn, Ni, and Zn. For area samples, the median concentrations of the above list were 0.459, 0.0875, 0.148, 2.54, 0.0571, 0.0711, and 1.19 µg/m^3^ at manufacturing sites, respectively. The same list at administrative sites were 0.132, 0.00761, 0.0156, 0.662, 0.0241, 0.00228, and 0.714 µg/m^3^. All seven metals showed significant differences between manufacturing and administrative sites.

**Table 2:** Concentration (μg/m^3^) of manufacturing and administrative sites for both area and personal samples.

Sample type	Manufacturing (N = 70)					Administrative (N = 16)					p value
Metal	Mean	(SE)	Median	Min	Max	Mean	(SE)	Median	Min	Max	
Area											
Al	7.62	(3.09)	0.459	0.005	153	0.258	(0.10)	0.132	0.013	1.53	p < 0.001
Cr	0.716	(0.393)	0.0875	0.0003	27.1	0.0149	(0.0046)	0.00761	0.0003	0.0676	p < 0.001
Cu	22.8	(19.9)	0.148	0.002	1380	0.0634	(0.0368)	0.0156	0.0042	0.601	p < 0.001
Fe	15.6	(7.73)	2.54	0.11	509	0.691	(0.128)	0.662	0.050	1.56	p < 0.001
Mn	0.571	(0.232)	0.0571	0.0007	14.0	0.0298	(0.0061)	0.0241	0.0029	0.0792	p < 0.001
Ni	15.3	(6.79)	0.0711	0.00001	298	0.0257	(0.0149)	0.00228	0.0001	0.240	0.006
Zn	2.24	(0.37)	1.19	0.02	14.3	0.664	(0.116)	0.714	0.038	1.23	0.018

	Manufacturing (N = 60)					Administrative (N = 15)					
Personal											
Al	7.59	(3.62)	0.504	0.026	211	0.271	(0.091)	0.157	0.016	1.46	p < 0.001
Cr	0.315	(0.084)	0.0863	0.0002	2.89	0.0178	(0.0058)	0.00725	0.00055	0.0783	p < 0.001
Cu	0.532	(0.160)	0.164	0.002	8.14	0.0674	(0.0245)	0.0305	0.0010	0.364	p < 0.001
Fe	29.7	(20.5)	2.57	0.48	1200	0.608	(0.113)	0.550	0.073	1.49	p < 0.001
Mn	0.798	(0.402)	0.0696	0.0012	22.5	0.0208	(0.0054)	0.0166	0.0019	0.0728	p < 0.001
Ni	0.758	(0.243)	0.0747	0.000003	10.8	0.0251	(0.0125)	0.00545	0.00009	0.180	0.015
Zn	1.74	(0.32)	1.09	0.04	12.7	0.638	(0.157)	0.516	0.041	1.75	0.035

For personal samples, the median concentrations of this list were 0.504, 0.0863, 0.164, 2.57, 0.696, 0.0747, and 1.09 µg/m^3^ at manufacturing sites. The concentrations at administrative sites were 0.157, 0.00725, 0.0305, 0.550, 0.0166, 0.00545, and 0.516 µg/m^3^ for the same list. Once again, these seven metals showed significant differences between both sites. This indicates that exposure assessment should focus exclusively on manufacturing sites to avoid dilution effects.

### Manufacturing-specific concentration comparison

The investigated plants comprised three types of manufacturing plants: surface treatment plants (7 plants), metal mold manufacturing plants (5 plants), and metal casting plants (3 plants). Metal die and mold manufacturing, and metal casting plants exhibited similar pollutant profiles characterized by metal dust and fumes generated during processes such as grinding, cutting, drilling, welding, and pressing of raw materials. The prevalent emission metals varied based on the composition of the workpieces and included Fe, Al, Zn, Cu, and Cr^8,40,41^. Therefore, these two types of plants were categorized as mold manufacturing/casting, where metal dust and fumes were the predominant particulate forms. Surface treatment procedures involve agitation of the electrolyte for efficient electroplating or anodizing, during which metal mists are generated. Zn, Ni, Cu, and Cr were commonly found in the mist due to the frequent usage of corresponding salts as electrolytes^10^. Because the administrative site was found to have distinct and lower levels, comparisons only included manufacturing site samples.

For area samples, the median concentrations of Al, Cr, Cu, Fe, Mn, Ni, and Zn were 1.06, 0.0746, 0.151, 4.86, 0.250, 0.00202, and 0.311 µg/m^3^ at mold manufacturing/casting plants. At surface treatment plants, the median concentrations of the same list were 0.405, 0.104, 0.145, 2.32, 0.0438, 0.394, and 1.67 µg/m^3^ (Table 3). Elevated levels of Al, Fe, and Mn, ranging from two to five times higher, were found in mold manufacturing/casting plants compared to surface treatment plants, with Fe and Mn showing significant differences between the two types of manufacturing plants. This difference might be attributed to machining on base metals, such as Fe and Mn, in metal molding and casting manufacturing.

**Table 3:** Concentration (μg/m^3^) of mold manufacturing/casting and electroplating plants for both area and personal samples.

Metal	Mean	(SE)	Median	Min	Max	Mean	(SE)	Median	Min	Max	p value
Sample type	Mold manufacturing/casting (N = 31)					Surface treatment (N = 39)					
Area											
Al	15.8	(6.7)	1.06	0.005	153	1.15	(0.58)	0.405	0.087	22.8	0.178
Cr	0.434	(0.191)	0.0746	0.0009	4.60	0.940	(0.690)	0.104	0.0002	27	0.347
Cu	0.436	(0.148)	0.151	0.002	4.18	40.5	(35.6)	0.145	0.034	1380	0.543
Fe	28.7	(17.0)	4.86	0.11	509	5.27	(2.53)	2.32	0.01	101	0.048
Mn	1.22	(0.50)	0.250	0.001	14.0	0.0513	(0.0061)	0.0438	0.0001	0	p < 0.001
Ni	0.0713	(0.0276)	0.00202	0.00001	0.813	27.5	(11.9)	0.394	0.0003	299	p < 0.001
Zn	0.882	(0.297)	0.311	0.017	9.06	3.32	(0.56)	1.67	0.50	14.3	p < 0.001

	Mold manufacturing/casting (N = 32)					Surface treatment (N = 28)					
Personal											
Al	12.8	(6.6)	1.67	0.03	211	1.68	(1.14)	0.452	0.228	32.3	0.208
Cr	0.437	(0.149)	0.0768	0.0002	2.89	0.175	(0.051)	0.0962	0.0069	1.20	0.625
Cu	0.648	(0.268)	0.165	0.002	8.14	0.399	(0.157)	0.163	0.060	4.43	0.801
Fe	53.0	(38.2)	4.62	0.48	1200	3.08	(0.44)	2.17	0.60	11.3	0.106
Mn	1.45	(0.74)	0.186	0.001	22.5	0.0525	(0.0065)	0.0473	0.0127	0.174	p < 0.001
Ni	0.159	(0.104)	0.0317	0.000003	3.37	1.44	(0.48)	0.298	0.006	10.8	p < 0.001
Zn	0.877	(0.331)	0.364	0.038	10.8	2.73	(0.51)	1.99	0.081	12.7	p < 0.001

On the other hand, Cr, Ni, and Zn were higher in electroplating plants, ranging from 1.5 to 200 times higher. These metals are commonly used as surface coating materials in electroplating to prevent corrosion of the base metal or for decorative purposes. Ni and Zn showed significant differences between these two types of manufacturing. This indicates that Ni-electroplating and Zn-electroplating were common processes in the investigated surface treatment plants, while these two metals had limited usage in metal mold manufacturing/casting.

Regarding personal samples, the median concentrations of Al, Cr, Cu, Fe, Mn, Ni, and Zn were 1.67, 0.0768, 0.165, 4.62, 0.186, 0.0317, and 0.364 µg/m^3^ in mold manufacturing/casting plants. At surface treatment plants, the median concentrations of the same list were 0.452, 0.0962, 0.163, 2.17, 0.0473, 0.298, and 1.99 µg/m^3^ (Table 3). Al, Fe, and Mn showed higher concentrations in mold manufacturing/casting plants, ranging from two to four times higher, with only Mn showing a significant difference. Cr, Ni, and Zn were at higher concentrations in surface treatment plants, ranging from 1.3 to 5 times higher, with Ni and Zn showing significant differences between mold manufacturing/casting and surface treatment plants, consistent with findings from area samples.

Notably, one of the surface treatment plants had extremely high Cr and Cu mean concentrations in area samples compared to personal samples. This raises concerns about the relationship between area and personal samples, the representativeness of area samples for personal exposure assessment, and the diversity in metal concentration in the investigated facilities. This finding suggests that it is preferable to use personal data for exposure assessment whenever possible.

### Exposure assessment of the investigated manufactures

Total concentrations, STLVr, and HI were calculated to assess the co-exposure to multiple metals. The TLV values and reference doses used in this study are shown in Table S3 (Supplemental information). Notably, values of Cr^3+^ and Cr^6+^ differed due to their different toxicities. Cr^3+^ was present in metal mold manufacturing/casting facilities, while Cr^6+^ was present in surface treatment facilities, reflecting the materials and chemicals used in each plant. Accordingly, the related values of Cr^3+^ and Cr^6+^ were 3 and 0.2 µg/m^3^ for TLV, and 0 and 0.002 µg/m^3^ for reference doses.

The results in area samples showed that total concentrations, STLV_r_, and HI were in the ranges of 3.01–348 µg/m^3^, 0.01–27.6, and 1.13–7790, respectively (Table 4). The three facilities with high total concentrations, in descending order, were plant 10 (electroplating plant), plant 6 (metal casting plant), and plant 14 (anodizing plant). High STLV_r_ were observed at plants 10, 13, and 9, all of which were electroplating plants. The three plants with high HI were 10, 14, and 13, which are electroplating, anodizing, and electroplating plants, respectively. Plant 10 ranked first among the three categories due to higher concentrations of Cr, Cu, and Ni. Plant 6 ranked second in the total concentration category due to a higher concentration of Fe. Plants 9 and 13 had characteristic emissions of Cr, while plant 14 had characteristic emissions of Ni.

**Table 4:** Mean concentrations of area samples, and corresponding ratios to ACGIH TLV and California chronic inhalation reference dose.

Metal		Mean concentration (μg/m^3^)														
		Mold manufacturing/casting								Surface treatment						
		Plant1	Plant2	Plant3	Plant4	Plant5	Plant6	Plant7	Plant8	Plant9	Plant10	Plant11	Plant12	Plant13	Plant14	Plant15
Al		0.37	38.3	42.2	1.67	31.1	10.8	0.16	0.24	1.99	0.39	0.24	0.42	0.73	3.59	0.28
Cr		V < 0.01^e^	0.06	2.42	0.03	0.26	0.51	0.03	0.16	0.31	5.11	0.06	0.03	0.47	0.06	0.12
Cu		0.07	0.04	0.15	0.08	1.04	1.92	0.17	0.37	0.41	262	0.10	0.13	0.21	0.22	0.07
Fe		10.8	1.91	4.83	7.10	4.82	240	1.41	11.5	4.71	1.91	1.01	1.35	5.17	16.6	2.58
Mn		0.24	0.50	2.60	0.31	0.21	6.99	0.10	0.27	0.14	0.03	0.04	0.04	0.05	0.05	0.03
Ni		V < 0.01	0.03	0.12	V < 0.01	0.06	0.04	V < 0.01	0.30	0.23	73.2	V < 0.01	0.93	0.46	89.2	V < 0.01
Zn		0.08	2.32	0.14	0.57	0.55	0.91	1.13	1.32	5.97	5.41	1.66	1.30	5.61	0.94	2.65
Sum		11.5	43.1	52.5	9.77	38.1	261	3.01	14.1	13.8	348	3.09	4.20	12.7	111	5.72
TLV^a^ (μg/m^3^)		Corresponding ratio to ACGIH TLV														
Al	1000	V < 0.01	0.04	0.04	V < 0.01	0.03	0.01	V < 0.01	V < 0.01	V < 0.01	V < 0.01	V < 0.01	V < 0.01	V < 0.01	V < 0.01	V < 0.01
Cr	0.2 or 3^b^	V < 0.01	0.02	0.81	V < 0.01	0.09	0.17	0.01	0.05	1.54	25.6	0.28	0.16	2.36	0.32	0.60
Cu	200	V < 0.01	V < 0.01	V < 0.01	V < 0.01	V < 0.01	V < 0.01	V < 0.01	V < 0.01	V < 0.01	1.31	V < 0.01	V < 0.01	V < 0.01	V < 0.01	V < 0.01
Fe	5000	V < 0.01	V < 0.01	V < 0.01	V < 0.01	V < 0.01	0.05	V < 0.01	V < 0.01	V < 0.01	V < 0.01	V < 0.01	V < 0.01	V < 0.01	V < 0.01	V < 0.01
Mn	100	V < 0.01	V < 0.01	0.03	V < 0.01	V < 0.01	0.07	V < 0.01	V < 0.01	V < 0.01	V < 0.01	V < 0.01	V < 0.01	V < 0.01	V < 0.01	V < 0.01
Ni	100	V < 0.01	V < 0.01	V < 0.01	V < 0.01	V < 0.01	V < 0.01	V < 0.01	V < 0.01	V < 0.01	0.73	V < 0.01	V < 0.01	V < 0.01	0.89	V < 0.01
Zn	2000	V < 0.01	V < 0.01	V < 0.01	V < 0.01	V < 0.01	V < 0.01	V < 0.01	V < 0.01	V < 0.01	V < 0.01	V < 0.01	V < 0.01	V < 0.01	V < 0.01	V < 0.01
Sum		0.01	0.07	0.88	0.02	0.13	0.31	0.01	0.06	1.56	27.6	0.29	0.17	2.37	1.22	0.60
CIR dose^c^ (μg/m^3^)		Corresponding ratio to California reference dose														
Cr	0.002 or –^d^									154	2.560	28.5	15.6	236	31.6	59.6
Mn	0.09	2.71	5.58	28.9	3.48	2.33	77.7	1.11	3.01	1.51	0.369	0.439	0.481	0.533	0.552	0.344
Ni	0.014	0.55	2.04	8.58	0.43	4.27	2.6	0.02	21.5	16.5	5.230	0.0	66.1	33.0	6.370	0.0
Sum		3.27	7.62	37.5	3.90	6.60	80.3	1.13	24.5	172	7790	28.9	82.1	270	6400	60.0

^a^ACGIH Threshold limit value, ^b^0.2 µg/m^3^ for Cr^6+^, 3 µg/m^3^ for Cr^3+^, ^c^California chronic inhalation reference dose, ^d^0.002 µg/m^3^ for Cr^6+^, none for Cr^3+^, ^e^Value < 0.01.

Regarding personal samples, the total concentration, STLV_r_, and HI were in the range of 3.59–404 µg/m^3^, 0.02–2.00, and 1.31–416, respectively (Table 5). The three facilities with high total concentrations, in descending order, were plants 6, 3, and 5, belonging to casting, mold manufacturing, and casting plants, respectively. In contrast, plants 9, 10, and 13 had high STLV_r_, all of which were electroplating plants. High HI was found at two electroplating plants (plants 10 and 13) and one anodizing plant (plant 14). The top three ranked plants for total concentration were metal mold manufacturing/casting, due to the abundance of base metals, such as Al, Fe, and Mn. As for the STLV_r_ and HI categories, the top three plants in the STLV_r_ and HI categories were surface treatment facilities with characteristic emissions of Cr and Ni.

**Table 5:** Mean concentrations of personal samples, and corresponding ratios to ACGIH TLV and California chronic inhalation reference dose.

Metal		Mean concentration (μg/m^3^)														
		Mold manufacturing/casting								Surface treatment						
		Plant1	Plant2	Plant3	Plant4	Plant5	Plant6	Plant7	Plant8	Plant9	Plant10	Plant11	Plant12	Plant13	Plant14	Plant15
Al		0.78	4.77	60.5	2.90	26.2	6.55	0.17	0.19	0.95	0.39	0.43	0.39	0.72	8.47	0.42
Cr		0.06	0.17	2.17	0.04	0.18	0.77	0.05	0.05	0.20	0.39	0.06	0.03	0.31	0.08	0.14
Cu		0.08	0.64	0.22	0.05	0.86	2.76	0.31	0.26	0.22	1.78	0.12	0.28	0.18	0.08	0.13
Fe		16.4	1.11	5.20	6.50	4.07	386	1.81	2.91	2.88	1.60	1.84	1.36	6.57	2.66	4.64
Mn		0.35	0.06	2.38	0.25	0.27	8.08	0.12	0.09	0.12	0.02	0.05	0.04	0.05	0.05	0.05
Ni		0.12	0.87	0.05	V < 0.01^e^	0.05	0.05	V < 0.01	0.12	0.13	2.27	V < 0.01	1.82	0.61	5.24	V < 0.01
Zn		0.24	2.79	0.22	0.53	0.50	0.21	1.14	1.38	3.32	1.62	2.66	1.33	6.87	0.80	2.51
Sum		18.1	10.4	70.7	10.3	32.1	404	3.59	5.01	7.82	8.07	5.16	5.26	15.3	17.4	7.88
TLV^a^ (μg/m^3^)		Corresponding ratio to ACGIH TLV														
Al	1000	V < 0.01	V < 0.01	0.06	V < 0.01	0.03	V < 0.01	V < 0.01	V < 0.01	V < 0.01	V < 0.01	V < 0.01	V < 0.01	V < 0.01	V < 0.01	V < 0.01
Cr	0.2 or 3^b^	0.02	0.06	0.72	0.01	0.06	0.26	0.02	0.02	1.01	1.97	0.31	0.16	1.55	0.41	0.69
Cu	200	V < 0.01	V < 0.01	V < 0.01	V < 0.01	V < 0.01	0.01	V < 0.01	V < 0.01	V < 0.01	V < 0.01	V < 0.01	V < 0.01	V < 0.01	V < 0.01	V < 0.01
Fe	5000	V < 0.01	V < 0.01	V < 0.01	V < 0.01	V < 0.01	0.08	V < 0.01	V < 0.01	V < 0.01	V < 0.01	V < 0.01	V < 0.01	V < 0.01	V < 0.01	V < 0.01
Mn	100	V < 0.01	V < 0.01	0.02	V < 0.01	V < 0.01	0.08	V < 0.01	V < 0.01	V < 0.01	V < 0.01	V < 0.01	V < 0.01	V < 0.01	V < 0.01	V < 0.01
Ni	100	V < 0.01	V < 0.01	V < 0.01	V < 0.01	V < 0.01	V < 0.01	V < 0.01	V < 0.01	V < 0.01	0.02	V < 0.01	0.02	V < 0.01	0.05	V < 0.01
Zn	2000	V < 0.01	V < 0.01	V < 0.01	V < 0.01	V < 0.01	V < 0.01	V < 0.01	V < 0.01	V < 0.01	V < 0.01	V < 0.01	V < 0.01	V < 0.01	V < 0.01	V < 0.01
Sum		0.03	0.08	0.81	0.02	0.09	0.43	0.02	0.02	1.01	2.00	0.32	0.18	1.57	0.47	0.70
CIR dose^c^ (μg/m^3^)		Corresponding ratio to California reference dose														
Cr	0.002 or –^d^									101	197	31.3	16.0	155	41.2	69.4
Mn	0.09	3.93	0.67	26.4	2.79	2.99	89.8	1.29	1.04	1.3	0.3	0.5	0.5	0.5	0.5	0.5
Ni	0.014	8.50	62.2	3.83	0.02	3.56	3.86	0.02	8.76	9.6	162	0.02	130	43.7	374	0.0
Sum		12.4	62.9	30.2	2.81	6.55	93.6	1.31	9.80	111	359	31.8	146	199	416	70.0

^a^ACGIH Threshold limit value, ^b^0.2 µg/m^3^ for Cr^6+^, 3µg/m^3^ for Cr^3+^, ^c^California chronic inhalation reference dose, ^d^0.002 µg/m^3^ for Cr^6+^, none for Cr^3+^, ^e^Value < 0.01.

Except for an extremely high area concentration of Cu in plant 10 and of Ni in plant 14, the metals Al, Fe, and Zn contributed a considerable amount to the total concentration. These metals are considered less toxic. Regarding the STLV_r_ outcomes, plants 9, 10, 13, and 14 exceeded unity in either area or personal samples. As such, co-exposure to these metals might pose a health hazard for these facilities. The contributing metals to STLV_r_ were Cr for plants 9, 10, and 13; and Ni for plant 14. Cr and Ni demonstrated chronic health effects in the respiratory system, immune system, skin, and had cancer potentials in the lung^15,16^. HI results showed that all investigated plants exceeded unity in both area and personal samples. Surface treatment plants had higher HI values than metal mold manufacturing/casting plants.

### Hazard contributing metals in the investigated plants

The application of TLV_r_ and HQ can identify hazard-contributing metals. Accordingly, the percent contribution of each metal to STLV_r_ was estimated. Attention and control should be directed towards metals exhibiting a high percentage contribution and corresponding TLV_r_ values surpassing unity.

As for area samples, the main contributing metals in mold manufacturing/casting plants were quite diverse. These included Al (0.4–58.5%), Cr (23.1–91.9%), Fe (0.1–30.6%), and Mn (1.6–34.7%). None of the TLV_r_ and STLV_r_ values exceeding unity in mold manufacturing/casting plants indicated that these workplaces can be considered safe for workers in terms of TLV value. On the other hand, the two main metals in surface treatment plants were Cr (26.0–99.5%) and Ni (0.0–73.3%). Several electroplating facilities (plants 9, 10, and 13) had TLVr values for Cr exceeding unity. Thus, there appears to be a need for control measures of Cr in surface treatment plants, as already noted above.

As for personal samples, similar results were found. The main metals were Al (0.8–27.9%), Cr (58.8–89.2%), Fe (0.1–17.8%), and Mn (0.8–18.6%) in mold manufacturing/casting plants. Again, none of the TLV_r_ and STLV_r_ values exceeded unity in this type of manufacturing. The main contributing metals for surface treatment plants were Cr (86.8–99.5%) and Ni (0.0–11.0%). Similar to the results of area samples, plants 9, 10, and 13, which were all surface treatment plants, explicated the TLV_r_ value of Cr exceeding unity. Again, it indicates that Cr should be subjected to control measures in surface treatment plants.

Comparison between area and personal samples revealed lower TLV_r_ values in personal samples. For instance, TLV_r_ values of personal samples for Cr, Cu, and Ni at plant 10, and Ni at plant 14 were lower compared to area samples. This could be attributed to the mobility of workers within the workplace. Manufacturing processes in the investigated plants were semi-automatic, meaning workers didn't remain in the most polluted areas. In contrast, area samples were usually positioned in polluted areas (or main processing sites) during the study to assess potential TLV_r_ exceedance. Consequently, personal sample results exhibited lower concentrations than area samples^42^.

TLV_r_ levels may also reflect characteristic emissions for each plant. For instance, higher TLV_r_ values of Al, Fe, and Mn were observed in mold manufacturing/casting plants compared to surface treatment plants, as these metals served as base materials for casting and metal mold manufacturing. Additionally, TLV_r_ values of metals within a single plant could depict distinct emission patterns. For example, in area samples, plant 10 (an electroplating plant) exhibited characteristic TLV_r_ values for Cr, Cu, and Ni at 25.6, 1.31, and 0.73, respectively (Table 4). A higher Cu ratio at plant 10 suggested involvement in several cupric electroplating processes such as copper cyanide electroplating, cupric sulfate electroplating, and cupric pyrophosphate electroplating. Similarly, another surface treatment plant (plant 14) involved in plasma chemical coating, hard anodizing, and electroless nickel coating displayed characteristic emissions of Ni and Cr with TLV_r_ values of 0.89 and 0.32 in area samples.

The HI for both area and personal samples ranged from 1.13 to 7790 (Table 4) and 1.31 to 416 (Table 5), respectively, and were assessed based on Cr, Mn, and Ni. All values exceeded unity, indicating the potential for causing chronic health effects. Prolonged exposure to these metals could lead to adverse effects on the kidneys, respiratory tract, hematologic system, immune system, and nervous system^43^. Main contributing metals in mold manufacturing/casting plants were Mn and Ni, with percentages ranging from 12.3 to 98.4% and 1.6 to 87.7%, respectively, in area samples, and from 1.1 to 99.3% and 0.7 to 98.9%, respectively, in personal samples. In surface treatment plants, Cr and Ni were the main contributing metals, with percentages ranging from 0.5 to 99.4% and 0.03 to 99.5%, respectively, in area samples, and from 9.9 to 99.2% and 0.03 to 90.0%, respectively, in personal samples. Ni had a higher percentage contribution in HI than in STLV_r_, since the reference dose of Ni was 0.014 μg/m^3^, significantly lower than the TLV (100 μg/m^3^). The HI results characterized the health potential and concern of dominant metals for each plant, providing crucial information for prioritizing hazardous material management and control measures once again.

### Incremental lifetime cancer risk (ILCR) of the investigated manufacturing plants

Among the investigated metals, cadmium and cadmium compounds, chromium (VI) compounds, nickel and nickel compounds are categorized as Group 1 by the International Agency for Research on Cancer (IARC). Cobalt metal, soluble cobalt (II) salt, cobalt (II) oxide, cobalt (II) sulfide, and other cobalt compounds are categorized as 2A, 2A, 2B, 3, and 3, respectively^44^. Since cancer risk is a non-threshold effect, the aforementioned metals detected in the investigated plants (not limited to ND rates < 50%) are used for cancer risk estimation. Metal speciation was taken into consideration for estimating cancer risk. Therefore, surface treatment plants and mold manufacturing/casting plants were considered to result in exposure to Cr(VI) and Cr metal/Cr(III), respectively, based on their raw material inventory. Cobalt species in the fabricated metal product industry mainly consist of Co metal, cobalt nitrate, and cobalt sulfate^45^, which belong to group 2A classified by the IARC. Accordingly, corresponding potency factors were applied. The incremental lifetime cancer risks for mold manufacturing/casting factories and electroplating factories were 0 to 5.30 × 10^–3^ and 8.09 × 10^–4^ to 6.85 × 10^–3^, respectively, (Table 6). Five of the mold manufacturing/casting plants had risks lower than the risk of 10^–4^^46^ (including one plant not showing any cancer risk from investigated metals). Surface treatment plants exhibited a higher cancer risk compared to manufacturing/casting plants, with all risks exceeding 10^–4^, indicating a high risk level. These findings align with previous studies reporting an increased risk of lung cancer^3,47^.

**Table 6:** Mean concentration, lifetime average daily dose and cancer risk of the investigated plants.

Metal		Mean concentration (μg/m^3^)														
		Mold manufacturing/casting								Surface treatment						
		Plant 1	Plant 2	Plant 3	Plant 4	Plant 5	Plant 6	Plant 7	Plant 8	Plant 9	Plant 10	Plant 11	Plant 12	Plant 13	Plant 14	Plant 15
Cd		0.0153	ND^b^	ND	ND	ND	ND	ND	ND	0.00302	ND	ND	ND	ND	ND	ND
Co		0.0407	5.77	0.0174	ND	ND	0.0800	0.372	0.142	0.0534	ND	ND	ND	0.00303	0.0503	0.0338
Cr		0.253	0.339	2.17	0.0590	0.177	0.765	0.0673	0.0546	0.201	0.393	0.0625	0.0426	0.311	0.0824	0.139
Ni		0.159	1.16	0.107	ND	0.0663	0.108	ND	0.123	0.134	2.27	ND	2.42	0.611	5.24	ND
PF^a^ (μg/kg-d)^–1^		LADD (lifetime average daily dose, μg/kg-d)														
Cd	0.015	0.000517	ND	ND	ND	ND	ND	ND	ND	0.000102	ND	ND	ND	ND	ND	ND
Co	0.027	0.00137	0.195	0.000588	ND	ND	0.00270	0.0126	0.00480	0.00181	ND	ND	ND	0.000102	0.00170	0.00114
Cr	0.51	0.00855	0.0115	0.0732	0.00199	0.00600	0.0259	0.00227	0.00185	0.00679	0.0133	0.00211	0.00144	0.0105	0.00278	0.00469
Ni	0.00091	0.00536	0.0393	0.00361	ND	0.00224	0.00364	ND	0.00414	0.00452	0.0768	ND	0.0818	0.0207	0.177	ND
		Cancer risk														
Cd		7.76 × 10^–6^	ND	ND	ND	ND	ND	ND	ND	1.53 × 10^–6^	ND	ND	ND	ND	ND	ND
Co		3.71 × 10^–5^	5.27 × 10^–3^	1.59 × 10^–5^	ND	ND	7.29 × 10^–5^	3.40 × 10^–4^	1.30 × 10^–4^	4.87 × 10^–5^	ND	ND	ND	2.77 × 10^–6^	4.59 × 10^–5^	3.08 × 10^–5^
Cr^+6^		ND	ND	ND	ND	ND	ND	ND	ND	3.47 × 10^–3^	6.78 × 10^–3^	1.08 × 10^–3^	7.35 × 10^–4^	5.35 × 10^–3^	1.42 × 10^–3^	2.39 × 10^–3^
Ni		4.88 × 10^–6^	3.57 × 10^–5^	3.29 × 10^–6^	ND	2.04 × 10^–6^	3.32 × 10^–6^	ND	3.77 × 10^–6^	4.11 × 10^–6^	6.99 × 10^–5^	ND	7.45 × 10^–5^	1.88 × 10^–5^	1.61 × 10^–4^	ND
Sum		4.97 × 10^–5^	5.30 × 10^–3^	1.92 × 10^–5^	NA^c^	2.04 × 10^–6^	7.63 × 10^–5^	3.40 × 10^–4^	1.34 × 10^–4^	3.52 × 10^–3^	6.85 × 10^–3^	1.08 × 10^–3^	8.09 × 10^–4^	5.38 × 10^–3^	1.63 × 10^–3^	2.42 × 10^–3^

^a^Inhalation potency factor; ^b^Non detected; ^c^Non applicable.

Except for one metal casting plant where the main cancer risk contributor was Ni, the main risk contributor of the other manufacturing/casting factories was Co, with a percentage range of 74.6% to 100%. Co arises from machining workpieces with Co content (milling, electrical discharge machining, cutting, hobbing, and chemical etching)^11^. Although the Co percentage in the workpiece would be low^8^, its potentially severe adverse health effects on the neurological, cardiovascular, and endocrine systems^48^ and potential carcinogenicity require careful consideration of Co exposure. The main cancer risk contributor of the surface treatment factories was Cr, with the percentage ranging from 87.3% to 100%. The findings of ILCR demonstrated the characteristic metals for different manufacturing categories and revealed surface treatment plants with higher cancer risks.

## Discussion

### Metal concentrations in the literature

Among the investigated metals, Cr and Ni, which have considerable health effect potential, were addressed and compared to previous studies. Additionally, several other studies investigating multiple metal exposure were noted and compared with our study. Table 7 presents a comparison of our study with previous ones. Cr exposure has been extensively studied in workplaces, especially in metal finishing, plating, and coating plants. The arithmetic mean (AM) concentration of total Cr in personal samples was 0.175 μg/m^3^ in surface treatment plants and 0.437 μg/m^3^ in mold manufacturing/casting plants in our study, which were lower than concentrations found in the workers’ breathing zones in the fabricated metal product industries in previous studies. For instance, Mäkinen et al. reported an AM of 10 μg/m^3^^49^, Pan et al. described a geometric mean (GM) of 35.65 μg/m^3^^50^, and Chen et al. found an AM of 3.0 μg/m^3^^7^ in electroplating plants. All these values were below the current Taiwan’s permissible exposure limit (PEL) and the US OSHA’s PEL of 1000 μg/m^3^ for total Cr (see Supplementary Information Table S2). Additionally, several studies reported Cr(VI) concentrations in either personal or area samples. For example, Pan et al.^50^ reported a GM of 19.45 μg/m^3^ for personal exposure in electroplating plants. Scarsselli et al.^51^ described an AM of 24.85 μg/m^3^ and 61.56 μg/m^3^ for male and female workers, respectively, in the manufacturing sector of fabricated metal products. The same study also examined area concentrations from different sectors, with AMs of 29.24 μg/m^3^ (N = 4045), 59.06 μg/m^3^ (N = 58), and 214.01 μg/m^3^ (N = 207) in the sectors of manufacturing fabricated metal products, construction, and manufacturing motor vehicles, respectively. Although the level of Cr(VI) in the fabricated metal product manufacturing sector was not the highest among industrial sectors, most Cr(VI) exposure (> 50%) occurred in this sector, indicating that the manufacture of fabricated metal products, with a large number of exposed workers, was a sector with considerable Cr(VI) exposure. Another study investigated Cr(VI) levels in processes such as steel passivation at a steel manufacturer, welding processes at an engineering shop, and electroplating processes at two hard chrome electroplating companies, with corresponding AM levels of 25, 4.6, and 4.9 μg/m^3^, respectively^52^. This reveals that information on manufacturing processes is also important for predicting contaminants in addition to the type of manufacturing.

**Table 7:** Comparison of metal concentrations, hazard quotient (HQ), hazard index (HI) and incremental lifetime cancer risk (ILCR) in this study with other available data.

First author, publication year, country	Metal									Sampling location/subject	Central tendency, particle size, sample size
	Al	Cr	Cr(VI)	Cu	Fe	Mn	Ni	Zn	HI		
Concentration (μg/m^3^)											
This study	1.68 (1.14)	0.175 (0.051)		0.399 (0.157)	3.08 (0.44)	0.0525 (0.0065)	1.44 (0.48)	2.73 (0.51)		Workers in surface treatment plants	AM (SD), T, 28
	12.8 (6.6)	0.437 (0.149)		0.648 (0.268)	53.0 (38.2)	1.45 (0.74)	0.159 (0.104)	0.877 (0.331)		Workers in mold manufacturing/casting plants	AM (SD), T, 32
Mäkinen et al. 2004, Finland		10 (10)								Workers in six electroplating shops	AM (SD), I, 29
Chen et al. 2014, China		3.0 (3.6)								Workers from three Cr-electroplating plants	AM, T, 72
Pan et al. 2018, Taiwan		35.65	19.45							Workers form 16 hard Cr-electroplating companies	GM, T, 105
Scarsselli et al. 2012, Italy			24.85 (0.001–1000)							Male workers in sector of fabricated metal products	AM (range), T, 3561
			61.56 (0.005–1000)							Female workers in sector of fabricated metal products	AM (range), T, 484
			29.24 (0.001–1000)							Sector of fabricated metal products	AM (range), T, 4045
			59.06 (0.045–100)							Construction sector	AM (range), T, 58
			214.0 (0.5–1000)							Sector of motor vehicles	AM (range), T, 207
Shaw et al. 2020, Canada			25 (0.02–89)							Near a steel passivation process at a steel manufacturer	AM (range), T, 7
			4.6 (4.1–4.9)							During stainless steel welding at an engineering shop	AM (range), T,4
			4.9 (0.01–9.3)							Near an electroplating bath at a Cr-electroplating shop	AM (range), T, 17
Chen et al. 2014, China							7.4 (6.4)			Workers from three Ni-electroplating plants	AM, T, 64
Scarsselli et al. 2018, Italy							25.63 (2–13)			Male workers in sector of fabricated metal products	AM(IQR), T, 4705
							18.57 (2–11)			Female workers in sector of fabricated metal products	AM(IQR), T, 385
Beattie et al. 2017, Great Britain			4 (< 0.1–10)				10 (< 10–600)			Workers in electroplating plants	Median (range), I, 7, 14, 8^a^
Onat et al. 2020, Turkey		0.109 (0.038)		0.206 (0.173)		0.0733 (0.0448)	0.0813 (0.0895)			Near dipping baths of a metal finishing plant	AM (SD), T, 10
		0.072 (0.017)		0.145 (0.144)		0.046 (0.026)	0.065 (0.089)			Near dipping baths of a metal finishing plant	AM (SD), PM2.5, 10
Kim et al. 2021, Korea	541 (168)	99.1 (212)					12.7 (16.6)	6.94 (3.67)		Hard Al anodizing process at a surface treatment plant	AM (SD), T, 8
	33.6 (19.5)	6900 (10,400)					6.89 (4.95)	8.07 (5.38)		Cr-plating process at a surface treatment plant	AM (SD), T, 8
Miller et al. 2010, USA				83.0 (17.0)	34.0 (15.6)			5.5 (6.4)		Workers at the electro-refining floor of a silver refinery	AM (SD), T, 2
				8.5 (3.1)	9.8 (5.0)			1.0 (–)		Workers at the furnace floor of a silver refinery	AM (SD), T, 4
				12.7 (9.3)	3.0 (1.5)			2.0 1.5)		At the electro-refining floor of a silver refinery	AM (SD), T, 6
				7.3 (2.1)	3.1 (1.2)			1.1 (0.4)		At the furnace floor of a silver refinery	AM (SD), T, 8
Keyter et al. 2019, South Africa		6		30	1440	55	8			During the gouging process at metal workshops	AM, I, 10
		4.5		5.2	1090	110	2			During the lancing process at metal workshops	AM, I, 6
HQ and HI											
This study		87.2 (16.0–197)				0.6 (0.3–1.3)	103 (0.02–374)		191 (31.8–416)	Workers in surface treatment plants	AM (range), T, 28
						16.1 (0.7–89.8)	11.3 (0–62.2)		27.5 (1.3–93.6)	Workers in mold manufacturing/casting plants	AM (range), T, 32
Onat et al. 2020, Turkey		0.164				0.21	1.06		1.43	Near dipping baths of a metal finishing plant	AM, PM2.5, 10
ILCR											
This study		3.03 × 10^–3^					4.69 × 10^–5^			Workers in surface treatment plants	AM, T, 28
							6.63 × 10^–6^			Workers in mold manufacturing/casting plants	AM, T, 32
Onat et al. 2020, Turkey		6.54 × 10^–5^					1.86 × 10^–7^			Near dipping baths of a metal finishing plant	AM, PM2.5, 10

*AM* arithmetic mean, *SD* standard deviations, *GM* geometric mean, *T* total particles, *I* inhalable particles, *IQR* 25^th^–75th percentile.

^a^Sample size for Cr, Cr(VI) and Ni of 7,14 and 8, respectively.

Ni is one of the frequently occurring metals in the fabricated metal product manufacture, especially in surface treatment plants. Our results showed that the AMs of Ni for personal samples in surface treatment plants and mold manufacturing/casting plants were 1.44 and 0.159 μg/m^3^ (Table 7), respectively, which were lower than concentrations found in the workers’ breathing zones in the fabricated metal product industries in previous studies. For example, Chen et al. showed that the AM of Ni was 7.4 μg/m^3^ from three nickel electroplating plants in Shanghai, China^7^. Scarsselli et al.^53^ reported the AM levels of male and female workers in the manufacturing sector of fabricated metal products were 25.63 and 18.57 μg/m^3^, respectively. Again, all these values were below the current Taiwan’s permissible exposure limit (PEL) of 100 and the US OSHA’s PEL of 1000 μg/m^3^ for Ni (see Supplementary Information Table S2). Usually, Ni was concurrently present with Cr(VI), and it was estimated that 77% of workers were exposed to both metals^53^. Therefore, several other studies would report the concentrations of Ni and Cr(VI) (or Cr) simultaneously. For example, Beattie et al. reported that the median concentrations of Ni and Cr(VI) were 10 and 4 μg/m^3^, respectively, at 53 electroplating companies in Great Britain^54^.

Recently, more studies have investigated exposure concentrations of multiple metals to obtain more detailed results. A Turkish study investigated concentrations of Cr, Cu, Mn, and Ni near dipping baths of a metal finishing plant. The AM concentrations were 0.109, 0.206, 0.073, and 0.081 μg/m^3^, respectively^55^. A Korean study, which evaluated multiple heavy metal exposures in a surface treatment plant, found that processes of hard anodizing and chromium plating had the highest concentrations of Al and Cr. The AMs of Al, Cr, Ni, and Zn were 541, 99, 12.7, and 6.94 μg/m^3^ for the Al anodizing process; and 33.6, 6900, 6.89, and 8.07 μg/m^3^ for the Cr plating process^56^. A USA study, which characterized exposures to airborne metals in a silver refinery, reported that the mean (AM) personal concentrations of Cu, Fe, and Zn were 83.0, 34.0, and 5.5 μg/m^3^ in the electro-refining process, and 8.5, 9.8, and 1.0 μg/m^3^ in the metal-melting process, respectively. As for the area concentration, the mean (AM) area concentrations in the same order were 12.7, 3.0, and 2.0 μg/m^3^ in the electro-refining process, and 7.3, 3.1, and 1.1 μg/m^3^ in the metal-melting process, respectively^57^. A South African study investigated metal fume composition from gouging and lancing processes at metal workshops. The average concentrations of Cr, Cu, Fe, Mn, and Ni were 6, 30, 1440, 55, and 8 μg/m^3^ for the gouging process; and 4.5, 5.2, 1090, 110, and 2 μg/m^3^ for the lancing process. The type and magnitude of emitted fumes were related to the constituents of workpieces and consumable electrodes (wire/filament/rod) in the processes. This explained the higher concentration of Cu and Ni in the gouging process compared with the lancing process, since the electrodes used in gouging were copper-coated carbon graphite ones and might contain a scarce amount of Ni^58^. Our results showed that the mean personal concentrations of Al, Cr, Cu, Fe, Mn, Ni, and Zn were 12.8, 0.437, 0.648, 53.0, 1.45, 0.159, and 0.877 μg/m^3^ for mold manufacturing/casting plants, and 1.68, 0.175, 0.399, 3.08, 0.0525, 1.44, and 2.73 μg/m^3^ for surface treatment plants, respectively. All these results indicated that manufacturing type, workplace processes, and composition of workpieces had a great influence on metal concentrations. Our results were comparable with those reported in the Turkish study and lower than those found in the other studies.

### Health risk estimation of exposure to multiple metals in the literature

In real-world situations, people are exposed to multiple chemicals, making it more appropriate to estimate the health risks of multiple exposures. The additive method is typically applied for co-exposure to multiple chemicals when extensive mechanistic information is not available, and this method has been utilized in various research endeavors. For instance, a Turkish study investigated concentrations and corresponding health risks of Cr, Mn, and Ni in a metal finishing plant^55^. The mean concentrations of Cr, Mn, and Ni in PM2.5 were 0.072, 0.046, and 0.065 µg/m^3^, respectively, with corresponding HQs of 0.164, 0.21, and 1.06, resulting in a total HQ of 1.43. The HQ for Ni exceeded one and contributed to 74% of the total HQ. In our study, the mean (range) HQs for Cr, Mn, and Ni were 87.2 (16.0–197), 0.6 (0.3–1.3), and 103 (0.02–374), respectively, in the surface treatment (electroplating and anodizing) plants, with the mean (range) HI being 191 (31.8–416) (Table 7). The highest contributing metals were Cr in the Cr-plating process (54.7–99.2%) and Ni in the Ni-plating and anodizing processes (88.7%—90.0%). Since the reference concentration of Cr used in the Turkish study was 0.1 µg/m^3^, higher than that used in our study (0.002 µg/m^3^), it led to a smaller number of HQs and might have underestimated the health risk of Cr.

Regarding carcinogenic effects, the arithmetic means (AMs) of ILCRs for Cr and Ni in male workers were 6.54 × 10^–5^ and 1.18 × 10^–6^ in the Turkish study^55^, while our results revealed the AMs of cancer risk of 3.03 × 10^–3^ and 4.69 × 10^–5^, respectively (Table 7), in workers of surface treatment plants. Our results seemed to suggest higher health effect potential than the Turkish study. Nevertheless, both studies indicated that Cr and Ni are predominantly hazardous metals in electroplating and anodizing plants.

Additionally, our study utilized the TLV_r_ and the ratio of TLV_r_ to STLV_r_ to identify metals with potential health effects and the highest contributing metal species, employing a similar method to the HQ/HI approach. The results revealed Cr as the most concerning metal with TLV_r_ exceeding unity in several Cr-plating plants and a considerable percentage contribution (23.1–99.9% in area samples, 58.8–99.5% in personal samples) to STLV_r_. This approach proves quite useful in industrial hygiene practice, not only identifying the most adverse health concerning metals in a specific plant but also detecting the most polluted plants under our investigation".

### Application of chronic effect estimators

To our knowledge, this is the first study to estimate the health effects of multiple metal exposure in terms of total concentration, STLV_r_, HI, and lifetime cancer risk. Two key findings were observed.

Firstly, STLV_r_ and HI are much better estimates for non-carcinogenic health hazards compared to total concentration. Among these two estimators, STLV_r_ was found to be more informative than HI, as all target metals in this study had TLV values, whereas only Cr, Mn, and Ni had reference dose values used to calculate HI. However, HI estimates were more conservative than STLV_r_. All plants' HI values exceeded unity, indicating a potential chronic health effect on workers in these plants. This estimate is more favorable to worker health and provides alarming messages for implementing control measures in these plants. Nevertheless, both estimates showed good correlations with statistically significance, with values of 0.921 (*p* < *0.001*) for area samples and 0.789 (*p* < *0.001*) for personal samples, respectively (see Supplementary Information Table S6). These two estimators also provided information related to the concerned metals (Cr and Ni) and plants (surface treatment plants) with high adverse health effect potential. In practice, it is advisable to use both estimates for prioritizing control of metals and plants and improving the workplace environment.

Secondly, the best available estimate was used for cancer risk estimation. The summation of risks from individual metals was calculated and expressed as the total cancer risk for each plant. The cancer risks of metal mold manufacturing/casting plants and surface treatment plants ranged from 0 to 5.30 × 10^–3^ and 8.09 × 10^–4^ to 6.85 × 10^–3^, respectively. The contributing metals to these cancer risks were Co and Cr, respectively.

The resulting information could indicate the facilities with high adverse health potentials and also reveal the influential metals related to health effects. With this information, regulatory agencies can identify the most hazardous industrial sectors and materials, prioritize control measures, and minimize adverse health effect potentials. Industrial manufacturers can also use this information for prioritizing control measures to achieve the most cost-effective management of hazardous materials.

## Conclusions

Fifteen fabricated metal product plants were included in this study, comprising five metal mold manufacturing plants, three metal casting plants, and seven surface treatment plants. The study aimed to investigate metal concentration distribution and corresponding health potential in terms of total concentration, STLV_r_, HI, and cancer risk in these plants. Characteristic emissions of Al, Fe, and Mn were found in the metal mold manufacturing/casting plants, while emissions of Cr, Ni, and Zn were found in the surface treatment plants. STLV_r_ and HI were found to be more useful than total concentration in assessing health potential. The findings revealed that Cr and Ni were the primary metals contributing to health hazards in terms of STLV_r_, which was more informative compared to HI, as all target metals had TLV values. The HI values, mainly contributed to by Cr, Mn, and Ni, exceeded unity for all plants. This indicated that the HI method appeared to be more sensitive and conducive to health protection than the TLV_r_ method. Regarding cancer risk, the metal mold manufacturing/casting plants had a lower risk than the surface treatment plants, and the metals contributing to these risks for these two types of plants were Co and Cr, respectively. This study established a useful procedure for evaluating health hazards and cancer risk, and suggested hazard mitigation priorities for industrial sectors and facilities accordingly. By doing so, resources can be effectively allocated to hazard control and minimizing adverse health effects.

### Supplementary Information

Supplementary Information.

## Data Availability

The datasets generated during and/or analyzed during the current study are available from the corresponding author on reasonable request.
