# Supplementary material for: Investigation of metal concentration distribution and corresponding health exposure assessment of fabricated metal product manufacturers
Source: Sci Rep. 2024 Jun 13;14:13662. doi: 10.1038/s41598-024-64277-0 (PMC11176342; doi:10.1038/s41598-024-64277-0)
Supplement: Supplementary file 1 — Supplementary Information. [file 41598_2024_64277_MOESM1_ESM.docx]

**Supplementary Information**

**Investigation of metal concentration distribution and corresponding health exposure assessment of fabricated metal product manufacturers**

Basic information of Cr, Ni, and Mn

Chromium (Cr) finds extensive use in various industries owing to its hardness and high resistance to corrosion [1, 2]. These properties can be evaluated as follows. The hardness of Cr is estimated using the Mohs hardness scale, and the value of 8.5 out of 10, indicating relatively high hardness. The corrosion rate is directly proportional to the amount of current in electrochemical corrosion. Yu et al. conducted tests on four types of stainless steel with Cr content ranging from 11.5% to 24.0%, and they observed the lowest current density and a stable passivation layer at the highest Cr content, indicating Cr's good corrosion resistance [3].

The primary use of chromium is in the production of stainless steel, nonferrous alloys, and chrome plating. Other applications include tanning, dyeing, catalysts, surface treatments, and refractories [2, 4, 5]. Cr is a transition metal with common oxidation states of 0, III, and VI [1, 2]. Both Cr(III) and Cr(VI) are of significant health concern due to their common presence and adverse health effects. Cr (III) is considered less toxic compared to Cr (VI) based on animal studies. Few or no tumor incidences were found in rats or mice exposed to Cr (III) through intrapleural or intrabronchial studies, while tumor incidence was significantly different from control for Cr (VI) exposure found in murine experiments [1]. Additionally, several epidemiological studies have shown that inhalation of Cr (VI) can lead to nasal perforation, decreased respiratory function, and bronchial asthma in chromium platers [6-8]. Cr(VI) exposure has been associated with many adverse health problems, such as cancer, dermatitis, asthma, chronic bronchitis, hypertension, chromosomal abrasions, back pains, metabolic syndrome, changes in hemoglobin levels, and DNA damage in lymphocytes [9]. Cr(VI) has been classified by the International Agency for Research on Cancer (IARC) as Group 1, indicating it is a carcinogen to humans [1].

Nickel (Ni) is extensively utilized in industrial and commercial applications, including alloy production (such as stainless steel and nonferrous alloys), electroplating, nickel-cadmium battery manufacturing, coin production, pigments, and catalysts, owing to its resistance to high temperatures, corrosion, oxidation, and its excellent ductility [10-12]. The corrosion resistance properties have been demonstrated by various studies. For instance, Yang et al. investigated the corrosion kinetics of austenitic stainless steels, ferritic/martensitic steels, and nickel-based alloys, finding that nickel-based alloys experienced the least weight gain during the oxidation process [13]. Similarly, Oleksak et al. observed the oxidation surface of Fe–Cr and Fe–Ni–Cr-based alloys using microscopic and spectroscopic techniques, revealing a thinner oxidation layer in the Fe-Ni-Cr alloy compared to the Fe-Cr alloy [14]. Sun et al. examined ductility and corrosion characteristics with varying Ni contents, showing an increase in tensile strength (404 – 445 MPa) and a decrease in corrosion rate (0.1329 to 0.04 g/m2-h) with increasing Ni contents [15].

The widespread application of Nickel leads to environmental pollution at all stages of smelting, refining, manufacturing, recycling and disposal. The toxicity of nickel depends on various factors such as exposure level, duration of contact, exposure route, and nickel species [12]. Nickel is a common sensitizing agent and is responsible for allergic contact dermatitis, as indicated by positive dermal patch tests [10]. Additionally, several case reports have suggested that inhalation of nickel dust may lead to asthma, lung fibrosis, bronchitis, pneumoconiosis, cardiovascular diseases, and respiratory cancers [1, 12].

IARC has categorized soluble and insoluble nickel compounds as Group 1 (carcinogens to humans). This classification is supported by several epidemiological studies that have found higher respiratory cancer risks in nickel refinery workers, along with positive results from murine exposure to Ni subsulfide and Ni oxide through inhalation. Both human and animal studies consistently indicate that the carcinogenicity of nickel compounds is limited to respiratory tumors through the inhalation route [16]. Ni metal and alloys have been classified as Group 2B carcinogens (possibly carcinogenic to humans) based on four epidemiological studies showing no significant excess of respiratory cancers in workers predominantly exposed to metallic nickel or alloys, along with several animal studies with inconsistent results in tumor incidence. For the animal studies, four out of ten showed significant tumor incidence in treated groups [1, 12].

Manganese (Mn) is an essential nutrient for humans, playing a crucial role in normal immune function, bone growth, protein and energy metabolism, metabolic regulation, and free radical defense systems [17, 18]. However, excessive exposure to Mn through inhalation and ingestion can lead to adverse health effects. Numerous epidemiological studies have documented the health effects of chronic exposure to high levels of Mn in occupational settings such as mining, smelting, welding, battery manufacturing, and steel production [19, 20]. Additionally, accidental ingestion of large quantities of manganese, such as through exposure to high levels in drinking water, has been reported [21].

These adverse effects are dose-dependent and primarily affect the nervous system. Individuals exposed to Mn concentrations thousands of times higher than normal environmental levels have exhibited symptoms such as slow hand movements, loss of coordination and balance, forgetfulness, anxiety, or insomnia. Severe cases may lead to a condition known as manganism, characterized by symptoms including tremors, difficulty walking, and facial muscle spasms, particularly in workers exposed to concentrations millions of times higher than normal air levels [20]. In addition to its effects on the nervous system, Mn exposure can also impact the lungs. Lung toxicity often manifests as an inflammatory response and an increased susceptibility to infections, potentially resulting in manganic pneumonia [22].

Tables

## Table S1. Characteristics of selected manufacturing plants.

| Plant | Establishment time | No. of Employee | Category | Region |
| --- | --- | --- | --- | --- |
| Plant 1 | 1963 | 488 | Metal module manufacturing | Central |
| Plant 2 | 2007 | 432 | Metal module manufacturing | Central |
| Plant 3 | 2001 | 353 | Metal module manufacturing | Central |
| Plant 4 | 1976 | 25 | Metal module manufacturing | Southern |
| Plant 5 | 1988 | 23 | Metal module manufacturing | Southern |
| Plant 6 | 1998 | 125 | Metal casting | Central |
| Plant 7 | 1998 | 125 | Metal casting | Central |
| Plant 8 | 2002 | 93 | Metal casting | Central |
| Plant 9 | 2008 | 181 | Surface treatment | Southern |
| Plant 10 | 2000 | 28 | Surface treatment | Northern |
| Plant 11 | 2007 | 13 | Surface treatment | Northern |
| Plant 12 | 1981 | 24 | Surface treatment | Northern |
| Plant 13 | 2007 | 105 | Surface treatment | Southern |
| Plant 14 | 2002 | 126 | Surface treatment | Central |
| Plant 15 | 2000 | 25 | Surface treatment | Central |

## Table S2. Quality assurance/quality control data for air samples of ten target metals.

| Metal | Slope | Inter- ception | R-square | LOD (µg/l) | LOQ (µg/L) | MDL (µg/m^3^) | Recovery (%) | Blank  (µg/sample) |
| --- | --- | --- | --- | --- | --- | --- | --- | --- |
| Al | 1746.7 | 161.81 | 0.9999 | 0.124 | 0.408 | 2.43×10^-3^ | 101.3 | 0.551 (0.048)^a^ |
| Cd | 4664.3 | 106.82 | 0.9999 | 0.018 | 0.060 | 3.55×10^-4^ | 100.6 | ND^b^ |
| Co | 10977 | 481.82 | 0.9999 | 0.018 | 0.061 | 3.63×10^-4^ | 98.3 | ND |
| Cr | 7996.9 | 252.41 | 0.9999 | 0.016 | 0.054 | 3.20×10^-4^ | 99.1 | 0.183 (0.004) |
| Cu | 3198.4 | 332.5 | 0.9999 | 0.018 | 0.061 | 3.62×10^-4^ | 98.5 | 0.043 (0.006) |
| Fe | 0.0049 | 0.0033 | 0.9971 | 1.000 | 3.224 | 1.92×10^-2^ | 103.4 | 0.348 (0.011) |
| Mn | 11394 | 482.78 | 0.9999 | 0.011 | 0.036 | 2.14×10^-4^ | 101.8 | 0.020 (0.001) |
| Mo | 4443.4 | 193.02 | 0.9999 | 0.018 | 0.061 | 3.65×10^-4^ | 97.9 | 0.026 (0.001) |
| Ni | 0.0039 | 0.0008 | 0.9996 | 0.020 | 0.067 | 3.96×10^-4^ | 100.2 | 0.077 (0.009) |
| Zn | 0.1919 | 0.0033 | 0.9993 | 0.020 | 0.066 | 3.92×10^-4^ | 101.2 | 0.244 (0.006) |

^a^ Mean (SD)

^b^ Non detected

## Table S3. Regulatory limit, recommended limit, reference dose, unit risk and cancer potency of target metals in this study

|  |  | Regulatory limits & recommended limits | | | |  | Exposure assessment parameters from Cal/OSHA | | | | | | |
| --- | --- | --- | --- | --- | --- | --- | --- | --- | --- | --- | --- | --- | --- |
| Metal | Species/form (appearance) | Taiwan OSHA PEL^(a)^  (µg/m^3^) | US OSHA PEL^(b)^  (µg/m^3^) | Cal/OSHA PEL^(c)^  (µg/m^3^) | US NIOSH REL^(d)^  (µg/m^3^) | ACGIH  TLV^(e)^ (µg/m^3^) | Acute Inhalation (μg/m^3^) | 8-Hour Inhalation  (μg/m^3^) | Chronic Inhalation  (μg/m^3^) | Chronic Oral  (μg/kg-d) | Inhalation Unit Risk  (μg/m^3^)^-1^ | Inhalation Cancer  Potency  Factor  (μg/kg-d)^-1^ | Oral Slope Factor  (μg/kg-d)^-1^ |
| Aluminum | Al metal and insoluble compounds |  | 5000 (R) 15000 (T) | 5000 (R) 10000 (T) | 5000 (R) 10000 (T) | 1000 (R) |  |  |  |  |  |  |  |
| Cadmium | Cd and compounds | 50 |  | 5 | 2 (R)  10 (T) | 2 (R)  10 (T) |  |  | 0.02 | 0.5 | 0.0042 | 0.015 |  |
| Chromium | Cr metal and insoluble salts | 1000 | 1000 | 500 | 500 | 500 (I) |  |  |  |  |  |  |  |
|  | Cr+6 | 50 |  | 5 | 0.2 | 0.2 (I, TWA)  0.5 (I, STEL) |  |  | 0.200 | 20 | 0.15 | 0.51 | 0.0005 |
|  | CrO3 |  |  |  |  |  |  |  | 0.002 | 20 | 0.15 | 0.51 | 0.0005 |
|  | Cr+2, Cr+3 | 500 | 500 | 500 | 500 | 3 (I) |  |  |  |  |  |  |  |
| Cobalt | Co and inorganic compounds | 50 | 100 | 20 | 50 | 20 (I) |  |  |  |  | 0.0077 | 0.027 |  |
| Copper | Fume | 200 | 100 | 100 | 100 | 200 | 100 |  |  |  |  |  |  |
|  | Dusts & mists | 1000 | 1000 | 1000 | 1000 | 1000 | 100 |  |  |  |  |  |  |
| Manganese | Mn metal and inorganic compounds | 1000 | 5000 | 200 | 1000 | 20 (R)  100 (I) |  | 0.17 | 0.09 |  |  |  |  |
| Molybdenum | Soluble compounds | 5000 | 5000 | 500 |  | 500 (R) |  |  |  |  |  |  |  |
|  | Insoluble compounds |  | 15000 | 10000 |  | 3000 (R) 10000 (I) |  |  |  |  |  |  |  |
| Iron | Iron oxide | 10000 | 10000 | 5000 | 5000 | 5000 (R) |  |  |  |  |  |  |  |
| Nickel | Soluble compounds | 100 | 1000 | 50 | 15 | 100 (I) | 0.2 | 0.06 | 0.014 | 11 | 0.00026 | 0.00091 |  |
|  | Insoluble compounds | 1000 | 1000 | 100 | 15 | 200 (I) | 0.2 | 0.06 | 0.020 | 11 | 0.00026 | 0.00091 |  |
|  | Metal | 1000 | 1000 | 500 | 15 | 1500 (I) | 0.2 | 0.06 | 0.014 | 11 | 0.00026 | 0.00091 |  |
| Zinc | Zinc Chloride | 1000 | 1000 | 1000 | 1000 | 1000 |  |  |  |  |  |  |  |
|  | Zinc oxide | 5000 | 5000 | 5000 | 5000 | 2000 (R) |  |  |  |  |  |  |  |

Footnotes:

^(a)^ Taiwan Occupational Safety and Health Administration (OSHA) Permissible Exposure Limit (PEL)

^(b)^ US Occupational Safety and Health Administration (OSHA) Permissible Exposure Limit (PEL)

^(c)^ California Division of Occupational Safety and Health (Cal/OSHA) Permissible Exposure Limit (PEL)

^(d)^ National Institute for Occupational Safety and Health (NIOSH) Recommended Exposure Limit (REL)

^(e)^ American Conference of Governmental Industrial Hygienists (ACGIH) Threshold Limit Value (TLV)

Abbreviations: R: respirable particulate mass; T: total dust; I: inhalable particulate mass; TWA: time weighted average; STEL: short-term exposure limit

## Table S4. Concentration distribution of ten metals in area samples of fifteen fabricated metal product manufacturers (N=86).

| Metal | N^a^ | Censoring rate | Mean ± SD | GM | Maximum | Percentile (µg/m^3^) | | | | |
| --- | --- | --- | --- | --- | --- | --- | --- | --- | --- | --- |
|  |  |  | (µg/m^3^) | (µg/m^3^) | (µg/m^3^) | 5 | 25 | 50 | 75 | 95 |
| Al | 78 | 9% | 6.89±24.6 | 0.723 | 153 | <LOD | 0.171 | 0.387 | 1.09 | 39.8 |
| Cd | 10 | 88% | 0.0980±0.295 | 0.0447 | 0.938 | <LOD | <LOD | <LOD | <LOD | <LOD |
| Co | 32 | 63% | 2.24±12.4 | 0.0365 | 70.1 | <LOD | <LOD | <LOD | 18.4 | 69.4 |
| Cr | 69 | 20% | 0.729±3.31 | 0.102 | 27.1 | <LOD | 0.00943 | 0.0658 | 0.219 | 1.58 |
| Cu | 69 | 20% | 23.1±167 | 0.206 | 1380 | <LOD | 0.0318 | 0.0972 | 0.246 | 1.42 |
| Fe | 79 | 8% | 14.0±61.0 | 2.74 | 509 | <LOD | 0.985 | 2.18 | 4.87 | 34.4 |
| Mn | 78 | 9% | 0.518±1.84 | 0.0877 | 14.0 | <LOD | 0.0273 | 0.0511 | 0.138 | 3.42 |
| Mo | 23 | 73% | 7.73±36.8 | 0.0711 | 176 | <LOD | <LOD | <LOD | 0.0114 | 0.110 |
| Ni | 45 | 48% | 23.9±69.7 | 0.548 | 298 | <LOD | <LOD | 0.0267 | 0.382 | 135 |
| Zn | 78 | 9% | 2.14±2.91 | 1.04 | 14.3 | <LOD | 0.377 | 1.08 | 16.7 | 9.36 |

^a^ Valid number

## Table S5. Concentration distribution of ten metals in personal samples of fifteen fabricated metal product manufacturers (N=75).

| Metal | N^a^ | Censoring rate | Mean ± SD | GM | | Maximum | | Percentile (µg/m^3^) | | | | | | | | | |
| --- | --- | --- | --- | --- | --- | --- | --- | --- | --- | --- | --- | --- | --- | --- | --- | --- | --- |
|  |  |  | (µg/m^3^) | (µg/m^3^) | | (µg/m^3^) | | 5 | | 25 | | 50 | | 75 | | 95 | |
| Al | 72 | 9% | 6.38±25.7 | 0.796 | 211 | | 0.0498 | | 0.228 | | 0.443 | | 1.64 | | 25.6 | |  |
| Cd | 7 | 88% | 0.00262±0.00570 | 0.00679 | 0.0153 | | <LOD | | <LOD | | <LOD | | <LOD | | <LOD | |  |
| Co | 33 | 63% | 0.439±2.00 | 0.0453 | 11.5 | | <LOD | | <LOD | | <LOD | | 0.0267 | | 0.182 | |  |
| Cr | 59 | 20% | 0.324±0.654 | 0.105 | 2.89 | | <LOD | | 0.0098 | | 0.0546 | | 0.144 | | 2.12 | |  |
| Cu | 65 | 20% | 0.506±1.19 | 0.186 | 8.14 | | <LOD | | 0.0552 | | 0.137 | | 0.281 | | 2.55 | |  |
| Fe | 73 | 8% | 24.5±143 | 2.52 | 1200 | | 0.210 | | 1.05 | | 2.05 | | 4.75 | | 45.9 | |  |
| Mn | 67 | 9% | 0.719±2.95 | 0.0940 | 22.5 | | <LOD | | 0.0229 | | 0.0521 | | 0.168 | | 2.84 | |  |
| Mo | 21 | 73% | 0.0744±0.0678 | 0.0584 | 0.294 | | <LOD | | <LOD | | <LOD | | 0.0168 | | 0.146 | |  |
| Ni | 40 | 48% | 1.14±2.22 | 0.275 | 10.8 | | <LOD | | <LOD | | 0.0332 | | 0.180 | | 4.89 | |  |
| Zn | 71 | 9% | 1.60±2.28 | 0.780 | 12.7 | | <LOD | | 0.221 | | 0.899 | | 1.68 | | 6.93 | |  |

^a^ Valid number

## Table S6. Correlation between personal vs area groups, total concentration vs STLV_r_ or HI, and STLV_r_ vs HI (non-parametric)

| Item | Correlation | p-value |
| --- | --- | --- |
| Personal vs Area groups |  |  |
| Total concentration | 0.636 | 0.011 |
| STLV_r_ | 0.968 | p < 0.001 |
| HI | 0.943 | p < 0.001 |
| Total concentration vs STLV_r_ |  |  |
| Area samples | 0.471 | 0.076 |
| Personal samples | 0.268 | 0.334 |
| Total concentration vs HI |  |  |
| Area samples | 0.775 | p < 0.001 |
| Personal samples | 0.146 | 0.603 |
| STLV_r_ vs HI |  |  |
| Area samples | 0.921 | p < 0.001 |
| Personal samples | 0.789 | p < 0.001 |

Abbreviations: STLV_r_: summation of corresponding ratio to threshold limit value; HI: hazard index

References

1. IARC: IARC monographs on the evaluation of carcinogenic risks to humans-Chromium, Nickel and Welding. In*.*, vol. 49: International Agency for Research on Cancer, World Health Organization; 1990.

2. The Facts on Chromium [<https://sites.dartmouth.edu/toxmetal/more-metals/chromium-a-thoroughly-modern-metal/the-facts-on-chromium/>]

3. Yu Y, Shironita S, Souma K, Umeda M: Effect of chromium content on the corrosion resistance of ferritic stainless steels in sulfuric acid solution. Heliyon 2018; 4(11):e00958. <https://doi.org/10.1016/j.heliyon.2018.e00958>

4. Chromium [<https://pubs.usgs.gov/publication/ofr01381>]

5. Chromium [<https://en.wikipedia.org/wiki/Chromium>]

6. Haines AT, Nieboer E: Chromium hypersensitivity. In: *Chromium in the Natural and Human Environments.* Edited by Nriagu JO, Nieboer E. New York: John Wiley & Sons; 1988: 497-532.

7. Bovet P, Lob M, Grandjean M: Spirometric alterations in workers in the chromium electroplating industry. Int Arch Occup Environ Health 1977; 40(1):25-32. <https://doi.org/10.1007/bf00435514>

8. Lindberg E, Hedenstierna G: Chrome plating: symptoms, findings in the upper airways, and effects on lung function. Arch Environ Health 1983; 38(6):367-374. <https://doi.org/10.1080/00039896.1983.10545822>

9. Junaid M, Hashmi MZ, Malik RN, Pei DS: Toxicity and oxidative stress induced by chromium in workers exposed from different occupational settings around the globe: A review. Environ Sci Pollut Res 2016; 23(20):20151-20167. <https://doi.org/10.1007/s11356-016-7463-x>

10. Cempel M, Nikel G: Nickel: A Review of Its Sources and Environmental Toxicology. Polish J of Environ Stud 2006; 15(3):375-382.

11. Nickel [<https://en.wikipedia.org/wiki/Nickel>]

12. Genchi G, Carocci A, Lauria G, Sinicropi MS, Catalano A: Nickel: Human Health and Environmental Toxicology. Int J Environ Res Public Health 2020; 17(3). <https://doi.org/10.3390/ijerph17030679>

13. Yang L, Qian H, Kuang W: Corrosion Behaviors of Heat-Resisting Alloys in High Temperature Carbon Dioxide. Materials (Basel) 2022; 15(4). <https://doi.org/10.3390/ma15041331>

14. Oleksak RP, Addou R, Gwalani B, Baltrus JP, Liu T, Diulus JT, Devaraj A, Herman GS, Doğan ÖN: Molecular-scale investigation of the oxidation behavior of chromia-forming alloys in high-temperature CO2. npj Materials Degradation 2021; 5(1). <https://doi.org/10.1038/s41529-021-00194-1>

15. Sun Y, Hu S, Xiao Z, You S, Zhao J, Lv Y: Effects of nickel on low-temperature impact toughness and corrosion resistance of high-ductility ductile iron. Materials & Design 2012; 41:37-42. <https://doi.org/10.1016/j.matdes.2012.03.039>

16. Seilkop SK, Oller AR: Respiratory cancer risks associated with low-level nickel exposure: an integrated assessment based on animal, epidemiological, and mechanistic data. Regul Toxicol Pharmacol 2003; 37(2):173-190. <https://doi.org/10.1016/s0273-2300(02)00029-6>

17. Aschner JL, Aschner M: Nutritional aspects of manganese homeostasis. Mol Aspects Med 2005; 26(4-5):353-362. <https://doi.org/10.1016/j.mam.2005.07.003>

18. Keen CL, Ensunsa JL, Watson MH, Baly DL, Donovan SM, Monaco MH, Clegg MS: Nutritional aspects of manganese from experimental studies. Neurotoxicology 1999; 20(2-3):213-223.

19. Santamaria AB: Manganese exposure, essentiality & toxicity. Indian Journal of Medical Research 2008; 128(4).

20. Peres TV, Schettinger MR, Chen P, Carvalho F, Avila DS, Bowman AB, Aschner M: "Manganese-induced neurotoxicity: a review of its behavioral consequences and neuroprotective strategies". BMC Pharmacol Toxicol 2016; 17(1):57. <https://doi.org/10.1186/s40360-016-0099-0>

21. Bouchard MF, Sauve S, Barbeau B, Legrand M, Brodeur ME, Bouffard T, Limoges E, Bellinger DC, Mergler D: Intellectual impairment in school-age children exposed to manganese from drinking water. Environ Health Perspect 2011; 119(1):138-143. <https://doi.org/10.1289/ehp.1002321>

22. ATSDR: Toxicological Profile for Manganese. In*.* Edited by Services USDoHaH. Atlanta, Georgia; 2012.
